# Evaluation of Hepatoprotective Activity and Oxidative Stress Reduction of *Rosmarinus officinalis* L. Shoots Tincture in Rats with Experimentally Induced Hepatotoxicity

**DOI:** 10.3390/molecules26061737

**Published:** 2021-03-20

**Authors:** Irina Ielciu, Bogdan Sevastre, Neli-Kinga Olah, Andreea Turdean, Elisabeta Chișe, Raluca Marica, Ilioara Oniga, Alina Uifălean, Alexandra C. Sevastre-Berghian, Mihaela Niculae, Daniela Benedec, Daniela Hanganu

**Affiliations:** 1 Department of Pharmaceutical Botany, Iuliu Haţieganu University of Medicine and Pharmacy, 400010 Cluj-Napoca, Romania; irina.ielciu@umfcluj.ro; 2 Department of Clinic and Paraclinic Sciences, University of Agricultural Sciences and Veterinary Medicine, 400372 Cluj-Napoca, Romania; raluca.marica@usamvcluj.ro; 3 PlantExtrakt, 407059 Cluj-Napoca, Romania; neliolah@yahoo.com (N.-K.O.); andreea.turdean@plantextrakt.ro (A.T.); 4 Department of Pharmaceutical Industry, Faculty of Pharmacy, Vasile Goldiş Western University of Arad, 310414 Arad, Romania; 5 Department of Pharmaceutical Chemistry, Faculty of Pharmacy, Vasile Goldiş Western University of Arad, 310414 Arad, Romania; c_elisabeta@yahoo.com; 6 Department of Pharmacognosy, Iuliu Haţieganu University of Medicine and Pharmacy, 400010 Cluj-Napoca, Romania; ioniga@umfcluj.ro (I.O.); dhanganu@umfcluj.ro (D.H.); 7 Department of Pharmaceutical Analysis, Faculty of Pharmacy, Iuliu Hațieganu University of Medicine and Pharmacy, 400349 Cluj-Napoca, Romania; alina.uifalean@umfcluj.ro; 8 Department of Physiology, Faculty of Medicine, Iuliu Haţieganu University of Medicine and Pharmacy, 400006 Cluj-Napoca, Romania; berghian.alexandra@umfcluj.ro; 9 Department of Clinical Sciences, Division and Infectious Diseases, University of Agricultural Sciences and Veterinary Medicine, 400374 Cluj-Napoca, Romania; mihaela.niculae@usamvcluj.ro

**Keywords:** *Rosmarinus officinalis* L., fresh young shoots tincture, polyphenols, terpenes, antioxidant, hepatoprotective

## Abstract

*Rosmarinus officinalis* L. is a widely known species for its medicinal uses, that is also used as raw material for the food and cosmetic industry. The aim of the present study was to offer a novel perspective on the medicinal product originating from this species and to test its hepatoprotective activity. The tested sample consisted in a tincture obtained from the fresh young shoots. Compounds that are evaluated for this activity are polyphenols and terpenoids, that are identified and quantified by HPLC–UV–MS and GC–MS. Antioxidant activity was assessed in vitro, using the DPPH, FRAP and SO assays. Hepatoprotective activity was tested in rats with experimentally-induced hepatotoxicity. In the chemical composition of the tincture, phenolic diterpenes (carnosic acid, carnosol, rosmanol, rosmadial) and rosmarinic acid were found to be the majority compounds, alongside with 1,8-cineole, camphene, linalool, borneol and terpineol among monoterpenes. In vitro, the tested tincture proved significant antioxidant capacity. Results of the in vivo experiment showed that hepatoprotective activity is based on an antioxidant mechanism. In this way, the present study offers a novel perspective on the medicinal uses of the species, proving significant amounts of polyphenols and terpenes in the composition of the fresh young shoots tincture, that has proved hepatoprotective activity through an antioxidant mechanism.

## 1. Introduction

Among the most important flowering plant families, the Lamiaceae family is one of the largest, comprising numerous species that are known for their biological activities or for their use in different branches related to economy [1,2,3]. Species belonging to the genera *Salvia* [4], *Melissa* [5] or *Thymus* [6] are the most well known, being widely used in different forms for the treatment of numerous pathologies, but also for their nutritional or economical values [4,5,6].

*Rosmarinus* is an important genus of the Lamiaceae family, comprising 2 species, *Rosmarinus eriocalyx* Jordan and Fourr. and *Rosmarinus officinalis* L., that are largely distributed in habitats from Southern and Northern Africa, Western Asia, Anatolia and the Mediterranean basin [7,8]. The most studied species is *Rosmarinus officinalis* L., rosemary, a worldwide cultivated plant, known for its culinary use and pharmacological properties that made it famous in traditional medicine. It has been reported that its leaves present significant therapeutic applications in managing a wide range of diseases such as diabetes mellitus, respiratory disorders, stomach problems and inflammatory diseases [9]. It is also used in the food industry, as a food flavoring and preservative due to its antioxidant and antimicrobial properties. *R. officinalis* is also used as raw material in cosmetic products [10].

Hepatic diseases are a main threat to public health, indicating problems to the hepatic tissue or to the liver functions, which can be caused by different factors, such as viruses or bacteria, autoimmune diseases, or by the external action of different chemicals (drugs or toxic compounds) [11,12]. Nowadays, modern medicine offers alternatives for the treatment of these pathologies, but despite the advances, few effective drugs that offer protection and regeneration of hepatic cells exist [13]. Moreover, existing treatments can cause adverse effects which make the therapy of these pathologies even harder [12]. Thereby, the need for identifying novel alternatives for the treatment of hepatic diseases and for the protection of the liver appears to be important, in order to develop novel agents with high efficiency and superior safety profile [11,12,13]. Mechanisms that underlie the hepatoprotective activity are strongly related to the capacity of antioxidants to scavenge reactive oxygen species (ROS) that are produced by the metabolic conversion of xenobiotics and induce oxidative stress and damage to the liver tissue [14].

Hepatoprotective activity is amongst the biological activities that is reported for *R. officinalis* extracts in a model of azathioprine-induced toxicity in rats [15], acetaminophen-induced liver damage [16,17], gentamicin-treated rats [18], hepatic damage induced by hypotermic-ischemia in rats [19] and alcoholic liver disease [20], being assigned to the essential oil composition [21] and to its composition in polyphenols [17,18,19], among which rosmarinic acid is the most representative [16]. Nevertheless, this biological activity is less documented and, to the best of our knowledge, there is no clear evidence on the compounds that are responsible for this activity. Moreover, there is also little evidence on the underlying mechanism of action, which is supposed to be due to the reduction of ROS [14,15]. On the other side, in vitro antioxidant activity of *R. officinalis* is largely studied, being reported to numerous vegetal products of the species: fresh aerial parts [9], flower extracts [7] or essential oil [10,22]. Compounds that are responsible for this activity are polyphenols [7], but also terpenes from the essential oil [10,22].

Rosmarinic acid (RA) is a phenolic compound firstly isolated from *Rosmarinus officinalis* L., having remarkable pharmacological activities. It is commonly found in Lamiaceae species, such as *Melissa* sp., *Salvia* sp., *Origanum* sp. or *Thymus* sp. Its pharmacological importance is mainly due to its antioxidant, anti-inflammatory, antimicrobial and anti-diabetic properties [23]. Its hepatoprotective activity is also reported and is assigned to an antioxidant mechanism [24]. Other polyphenols that are found in the composition of the species are caffeic acid, chlorogenic acid, *p*-coumaric acid, quinic acid, kaempferol, quercetin, rutin and apigenin [7,25]. Among the phenolic terpenes, those such as carnosol and carnosic acid are also reported to be found in the composition of the species [26,27,28]. Essential oil of *R. officinalis* is rich in a large variety of monoterpenes such as borneol, camphor, linalool, α-and β-pinene, camphene, 1,8-cineole [22,29,30,31].

In this context, the main objective of the present study was to evaluate the hepatoprotective activity of a fresh young shoots of *R. officinalis* tincture against carbon tetrachloride (CCl_4_)-induced hepatotoxicity in rats. Compounds that are analyzed for this activity are the phenolic compounds and terpenoids. Moreover, the present study aims to offer important details on the mechanism underlying this activity, which appears to be due to the antioxidant properties of the tested extract. Finally, the present study also aimed to prove the significant potential of fresh young shoots as an important source of compounds exhibiting antihepatotoxic activity by an antioxidant mechanism.

## 2. Results and Discussion

The tincture, obtained from fresh young shoots macerated with 90% *v/v* ethanol, was analyzed from the physico-chemical point of view, according to European Pharmacopoeia (EPh) and German Homeopathic Pharmacopoeia (GHPh). Thus, the tincture presented a dark brown color, being an aromatic liquid with a relative density of 0.920. The value of the dry residue was 2.38% and the ethanol content was 55% vol. The identity according to the GHPh was performed by Thin Layer Chromatography (TLC) highlighting the main terpenoidic compounds.

### 2.1. Phytochemical Analysis of Tincture

The tincture was analyzed by HPLC–UV–MS for the identification and quantification of phenolic compounds. Results were expressed as μg polyphenol/g dry vegetal material (d.w.) (Table 1).

High amounts of polyphenols were identified in the composition of the fresh young shoots tincture. Phenolic terpenes were found to be the majority class of compounds. Among these, the one that was found in the highest concentration was carnosic acid, followed by rosmadial, carnosol and rosmanol. Among flavones, luteolin-glucuronide, cirsimaritin and homoplantaginin were also found in significant amounts. Rosmarinic acid is the hydroxycinnamic acid that was found in the highest amounts. Another compound that was found in significant amounts is syringic acid, a hydroxybenzoic acid.

Polyphenolic compounds from *R. officinalis* were reported in scientific literature, but the raw material was in general represented by the leaves, not the young shoots, which emphasize even more the originality of the present study. Carnosic acid and carnosol, together with rosmarinic acid are found in the composition of the species, but their presence is cited in the composition of the leaves [26,28,32], that are mostly dried [26,28]. Luteolin-glucuronide is also the flavonoidic compound that is mostly cited in the composition of the same parts of the species [28,32,33]. Presence of cirsimaritin, rosmanol, rosmadial and homoplantaginin is also reported for the leaves [32]. Differences can be found in the amounts of these compounds, significantly higher in the composition of fresh young shoots. Moreover, the novelty of the present study consists nevertheless in the vegetal material that is studied, which is represented by fresh young shoots, which, to the best of our knowledge, was not previously studied. Taking into consideration the fact that high amounts of these polyphenols were found in the composition of the tested tincture, important correlation could be found with the tested biological activities.

**Figure 1:**
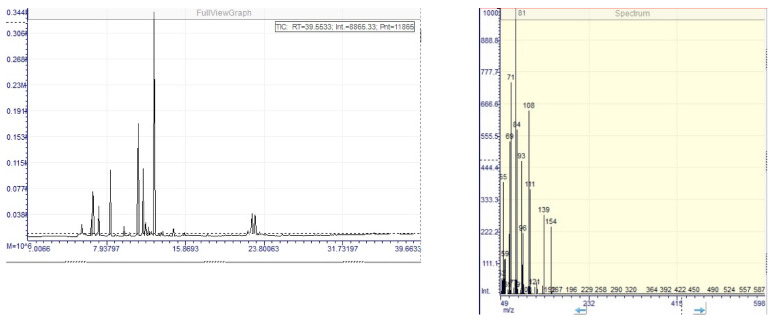
**Left**—GC chromatogram of *Rosmarinus officinalis* tincture. **Right**—MS spectra of separated 1,8-cineole.

In addition, terpenoids that are found in the essential oil were also found in significant amounts in the composition of the tested tincture, being solubilized by the extraction solvent, concentrated ethanol. The terpenoids were evaluated by a GC–MS method and the main components identified and quantified were 1,8-cineole, camphene, linalool, borneol, and terpineol (Figure 1-left). These compounds were also previously identified in the composition of the essential oil belonging to this species [7,14,31,34,35]. The antioxidant and hepatoprotective activities of the tested tincture can also be attributed to the presence of high percentage of 1,8-cineole (27.5 mg/100 g tincture), which is found to be the majoritary compound in the composition of the tested tincture (Figure 1-right). These compounds previously reported hepatoprotective activity [10,12,14] and the high amount that is found in the tested tincture, together with the phenolic compounds, may synergistically act in order to exert these biological activities. The obtained results of the GC–MS analysis are presented in Table 2.

### 2.2. Antioxidant Activity: In Vitro Assays

The antioxidant capacity was evaluated by three in vitro methods: 2,2-diphenyl-picrylhydrazil (DPPH), ferric-reducing antioxidant power (FRAP) and superoxid (SO) anion radical scavenging assay, assessing therefore by different mechanisms of the antioxidant capacity of the tested sample [36,37,38] (Table 3).

The total polyphenols, flavonoids and rosmarinic acids content of the *R. officinalis* tested tincture could be correlated with the antioxidant effects, as they showed significantly important amounts, compared to the dried leaves or extracts [39,40].

The significant content of polyphenolic compounds in the tested extract is highly related to its antioxidant potential. Synergistic, additive or antagonistic interactions between them can be revealed in this activity of neutralization of free radicals [41]. Most of the existing studies assign the antioxidant effect of the species to the essential oil content [22,31], but as high amounts of polyphenols are evaluated in different studies, it seems that they are very important for the antioxidant activity of the extracts [36].

The DPPH radical scavenging activity of the extract was high (IC_50_ = 31.85 ± 1.81 µg/mL). Previous studies have reported the antioxidant activity by the same method [7,40], testing the flowers [7] or the dried leaves [40] extracts. Other studies that were performed on fresh plants showed significantly higher value of IC_50_, indicating therefore a poorer effect of the whole mature plants [25].

The tested tincture has demonstrated a very good ferric ion-reducing antioxidant capacity with a value of 257.88 ± 1.74 µm TE/g, higher than other data revealed by other studies performed on leaves [26]. Antioxidant activity by FRAP method of *R. officinalis* extract was evaluated by Gîrd et al. as EC_50_ = 285.25 ± 0.88 µg/mL, but the study was performed on dried leaves [40].

In the case of superoxide (SO) scavenging assay, the decrease of absorbance at 560 nm with antioxidant compounds indicated the consumption of superoxide anion in the reaction mixture. The phenolic and terpenoid compounds of the tested tincture have the ability to neutralize superoxide radicals, which are extremely aggressive in liver tissue, causing significant cell damages. At the same time, superoxide radicals can be inhibited by the endogenous antioxidant enzyme, superoxide dismutase (SOD), whose level can be increased by the same compounds. In this way, the tested compounds synergistically act and help to link the antioxidant activity proven by in vitro experiments with those tested in vivo. The results of in vitro experiments show a good effect of removing superoxide radicals of *R. officinalis* fresh young shoots tincture, which was consistent with the in vivo results indicating an increase in SOD activity [42].

As it can be observed, the comparison with scientific literature is difficult, since different extraction methods and experimental protocols have been used and different plant material represented the starting point. However, taking into consideration all of the above, it becomes clearer that the fresh young shoots represent an important source of polyphenols and terpenes with promising antioxidant potential. Moreover, this antioxidant potential that is hereby proved is directly correlated with the hepatoprotective activity in vivo.

### 2.3. Hepatoprotective Activity: Animal Studies

The protective effect of *R. officinalis* extract was investigated using liver toxicity model induced by carbon tetrachloride (CCl_4_) (1 mL/kg).

The CCl_4_-hepatotoxicity model is extensively used to evaluate the hepatoprotective effects of drugs and plant extracts. It has been reported that one of the principal causes of CCl_4_-induced liver injury is the lipid peroxidation which is induced and accelerated by free radical derivatives of CCl_4_ [43]. CCl_4_ exerts its hepatotoxic effect through covalent binding of CCl_4_ metabolites and reaction with oxygen to initiate lipid peroxidation [44]. The extent of liver oxidative damage was investigated by measuring the level of malondialdehyde (MDA) and the activity of antioxidant enzymes as glutathione peroxidase (GPx), catalase (CAT) and superoxide dismutase (SOD). Liver injury was assessed by measuring of plasma activity of transaminases, as alanine aminotransaminase (ALT), aspartate aminotransaminase (AST), gamma-glutamyl transferase (GGT), while albumins reflected the liver function.

**Figure 2:**
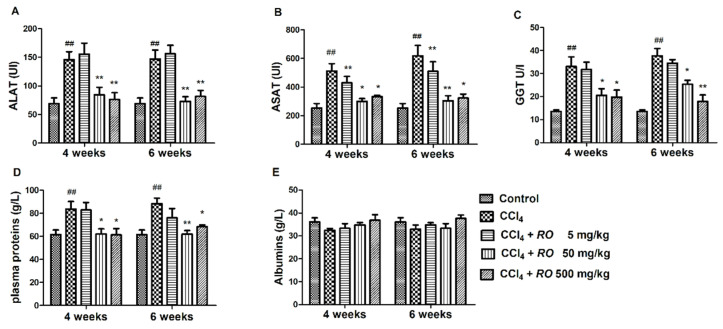
Effects of *R. officinalis* tincture on: alanine aminotransferase (ALAT) (**A**); aspartate aminotransferase (ASAT) (**B**); gamma glutamyl transferase (GGT) (**C**) activity and plasma total proteins (**D**); and albumins (**E**) concentration (mean ± SD, 5 animals/group). ## *p* < 0.01, compared to the Control group; * *p* < 0.05, ** and *p* < 0.01 compared to the CCl_4_ alone treated group.

All animals survived up to the end of the study; they showed no clinical signs and maintained a body weight gain, food and water consumption similar to control group. In mice receiving CCl_4_ and no therapy, the liver injury was reflected in elevated activity of plasma transaminases, ALT, AST, and GGT (Figure 2), but albumin levels maintained close to the values of the control group, suggesting the absence of liver failure. However, the total proteins were elevated, based on globulin fraction, probably as a result of liver inflammatory reaction. *R. officinalis* tincture administration revealed a protective effect, visible at the doses of 50 and 500 mg/kg b.w. However, the higher dose of 500 mg/kg b.w. did not provide a better protective effect, thus no dose-dependent effect has been found. When comparing the two time intervals, ASAT and GGT had a slightly increasing trend, but only the variation of ASAT was statistically relevant (*p* < 0.05), while ALAT remained at the same level. Similarly, the plasma proteins remained at the same levels. In the groups receiving therapy, the values remained also very similar.

**Figure 3:**
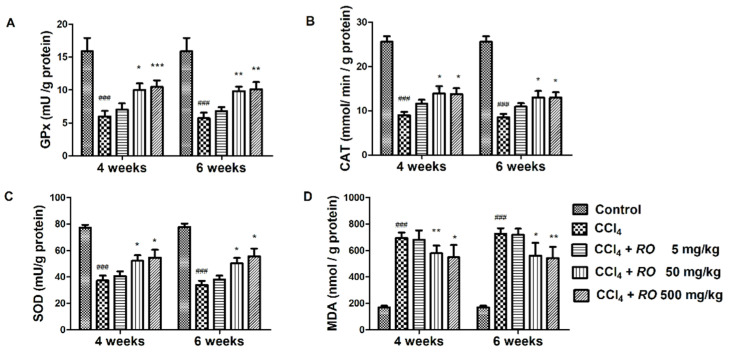
Effects of *R. officinalis* tincture on: glutathione peroxidase (GPx) (**A**); catalase (CAT) (**B**); superoxide dismutase (SOD) (**C**); and malondialdehyde (MDA) (**D**) (mean ± SD, 5 animals/group). ### *p* < 0.001, compared to the Control group; * *p* < 0.05, ** *p* < 0.01 and *** *p* < 0.001 compared to the CCl_4_ alone treated group.

The protective effect of *R. officinalis* extract seems to be exerted by antioxidant effect. Expectedly, CCl_4_ induced inhibition of antioxidant GPx, CAT, SOD and a threefold increase of MDA levels. The doses of 50 and 500 mg/kg b.w. of *R. officinalis* partially restored the activity of antioxidant enzymes and alleviated the lipid peroxidation. The values remained significantly altered compared to those of the control group. Once again, the higher dose of 500 mg/kg did not provide a better protection than the dose of 50 mg/kg did (Figure 3). In accordance to transaminase levels, the oxidative stress markers showed no significant variations between the two time intervals.

**Figure 4:**
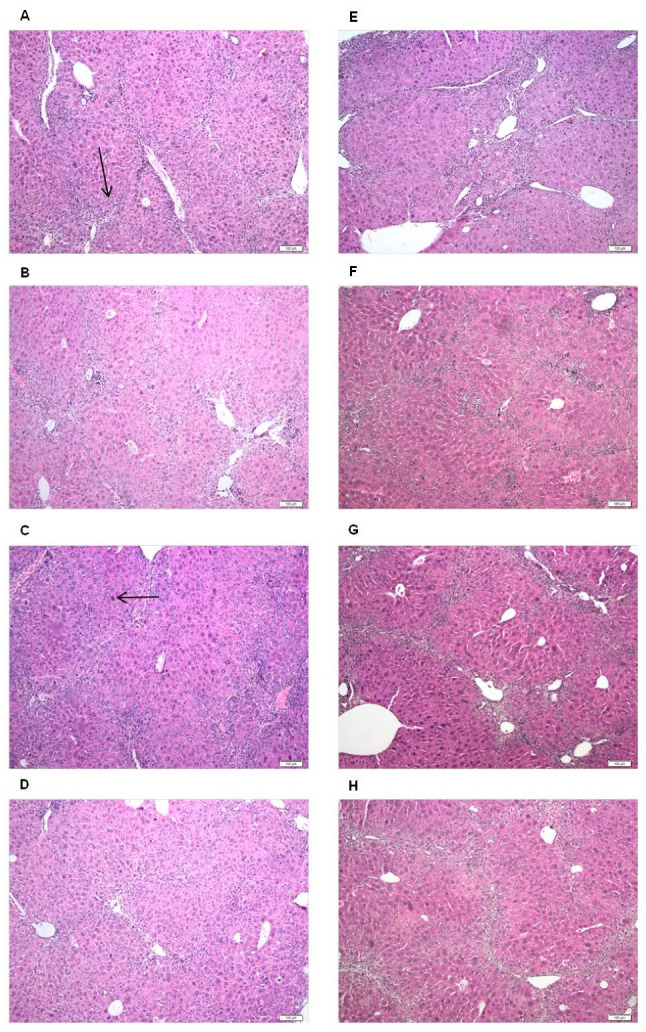
Effects of *R. officinalis* tincture on the histologic aspect of the liver: (**A**,**E**) the negative control groups receiving only carbon tetrachloride (1 mL/b.w.) showed inflammatory infiltrate (black arrow) and hepatocyte necrosis; the groups receiving therapy with the extract in a dose of: 5 mg/b.w., (**B**,**F**); 50 mg/b.w.; (**C**,**G**); and 500 mg/b.w. (**D**,**H**). Duration: (**A–D**) four weeks; (**E**–**H**) six weeks. Hematoxylin & Eosin stain; Bar, 100 μm.

Histological examination revealed necrosis and inflammatory cells infiltrate as the predominant features in the groups receiving CCl_4_, in absence of *R. officinalis* therapy. Hence, around portal spaces, most of the hepatic lobules exhibited multifocal hepatocyte necrosis surrounded by inflammatory infiltrate mainly composed of neutrophils and macrophages. Another important feature of hepatic toxicity was the proliferation of fibrous tissue between portal spaces. In the groups receiving *R. officinalis* therapy, we observed an improvement in the hepatic regeneration, dependent to the dose. The main effect of the extract was the downregulation of the inflammatory reaction; better manifested in the group receiving the dose of 50 mg/b.w. for 6 weeks long (Figure 4G). Although the inflammation was not quantitatively assessed, we noticed a marked decrease in the number of neutrophils and macrophages. Notably, this improvement was less obvious in the first (5 mg/b.w.) and the third groups (500 mg/b.w.), suggesting that, in high doses, the extract may exert a toxic effect on the liver or may interfere with the regeneration processes. The *R. officinalis* therapy improved also the regeneration process, manifested by the presence of mitotic figures, indicating the restitution of the hepatic architecture and function (Figure 4C). Reduction of the hepatocyte necrosis and fibrosis were also noticed, mainly for the dose of 50 mg/b.w. Once again, the benefits were minimal in the case of animals receiving the doses of 5, and 500 mg/b.w. (Figure 4D,H).

## 3. Materials and Methods

### 3.1. Chemicals and Reagents

Acetonitrile for the HPLC-gradient was provided by Merck (Darmstadt, Germany) and water was purified with a Direct-Q UV system by Millipore (Darmstadt, Germany). Luteolin was purchased from Sigma (Darmstadt, Germany) and all other chemicals used were obtained from Alfa-Aesar, Karlsruhe, Germany.

### 3.2. Plant Material, Preparation and Characterization of Fresh Young Shoots Tincture

The vegetal material was harvested from the ecological culture of PlantExtrakt (Rădaia, Cluj county, Romania), in September 2020. Voucher specimens are deposited in the herbarium of the Pharmacognosy Department of the Faculty of Pharmacy Cluj-Napoca (Voucher no. 159). The *R. officinalis* tincture was prepared according to the German Homeopathic Pharmacopoeia (GHPh) and European Pharmacopoeia (EPh) specifications for tincture preparation (method 1.1.5). Fresh, young shoots were extracted by cold maceration with 90% *v/v* ethanol. One part of the crushed vegetal material was mixed with 1.4 parts of ethanol for 10 days. Afterwards, the extract was filtered. For the physicochemical characterization of the obtained tincture, organoleptic properties, relative density, residue at evaporation and ethanol content were assessed according to the methods of the EPh [45]. Before use in the animal experiment, the tincture was evaporated until the complete removal of the alcohol and immediately given to animals.

The relative density was performed according to the European Pharmacopoeia (EPh) using a Mettler Toledo (Greifensee, Switzerland) digital densimeter. The dry residue was performed according to the European Pharmacopoeia (EPh) using Kern analytical scale (Berlin, Germany) and a Memmert drying cabinet (Schwabach, Germany). The ethanol content was assessed according to European Pharmacopoeia (EPh) using a Neo-Clevenger apparatus and a Mettler Toledo digital densimeter.

### 3.3. Quantification of Total Polyphenols, Flavonoids and Phenolic Acids Content

The total phenolic content (TPC) was evaluated by a spectrophotometric method using the Folin–Ciocâlteu reagent, according to the European Pharmacopoeia. TPC values were calculated using the calibration curve of gallic acid (R^2^ = 0.9931) and expressed as mg gallic acid equivalents (GAE)/g fresh vegetal material. Quantitative determination of flavonoids (TFC) was performed by a spectrophotometric method using aluminum chloride. TFC values were determined using an equation obtained from a calibration curve of rutoside (R^2^ = 0.9992) and expressed as mg of rutoside equivalents (RE)/g fresh vegetal material. Quantitative determination of phenolic acids (TPA) was analyzed by a spectrophotometrical method according to the 10th Edition of the Romanian Pharmacopoeia (*Cynarae folium* monograph). TPA results were expressed as mg rosmarinic acid equivalents (RAE)/g fresh vegetal material, calculated using a rosmarinic acid calibration curve graph (R^2^ = 0.9956). All experiments were performed in triplicate [46,47].

### 3.4. HPLC/DAD/ESI+ Analysis

This analysis was performed using a HP-1200 liquid chromatograph equipped with a quaternary pump, autosampler, DAD detector and MS-6110 single quadrupole API-electrospray detector (Agilent-Technologies Inc., Santa Clara, CA, USA). Positive ionization mode was used for the detection of phenolic compounds. Different fragmentors, in the range 50–100 V, were applied. The separation of compounds was carried out on an Eclipse XDB-C18 (5 μm; 4.5 × 150 mm i.d.) column (Agilent). Mobile phase consisted in water acidified with acetic acid 0.1% (A) and acetonitrile acidified with acetic acid 0.1% (B). Elution was performed in a multistep linear gradient, with the following composition: 5% B for 2 min; from 5% to 90% of B in 20 min, hold for 4 min at 90% B, then 6 min to arrive at 5% B. Flow rate was 0.5 mL/min and oven temperature 25 ± 0.5 °C. Detection of positively charged ions was performed by mass spectrometry, using the Scan mode and the following conditions: gas temperature 3500 °C, nitrogen flow 7 L/min, nebulizer pressure 35 psi, capillary voltage 3000 V, fragmentor 100 V and *m/z* 120–1200. Recording of chromatograms was performed at λ = 280 and 340 nm. The data acquisition was carried out with the Agilent ChemStation software [48,49].

### 3.5. GC–MS Analysis

For this analysis, a Dani Master GC–MS System, equipped with a SH-Rxi-5 ms column with 30 m × 0.25 mm × 0.25 µm was used. Nitrogen was used as carrier gas, with 10 mL/min flow rate and the temperature gradient in Table 4.

The EIS-MS detector was used to identify compounds with molecular weight from 50 to 600 daltons and the ion source was operated at 200 °C. Five µL tincture diluted 1 to 10 with absolute ethanol and 0.1 µL references having concentrations from 10 to 1280 µg/mL were injected. The terpenoids were identified based on the match factor (higher than 750), using the NIST MS 2.2 spectra database. The content of main terpenes was determined using the calibration curve method [45].

### 3.6. Antioxidant Activity Assays

#### 3.6.1. DPPH Radical Scavenging Activity

The first assay that was used is the DPPH bleaching assay, a spectrophotometric method based on the reaction of the DPPH reagent and antioxidants. Two mL of tincture at different concentrations were added to 2 mL of a DPPH methanolic solution at a concentration of 0.1 g/L and maintained at 40 °C in a thermostatic bath for 30 min. Changes in absorbance were measured at 517 nm and inhibition of the DPPH radical was calculated using the following formula: DPPH scavenging ability % = (A control − A sample/A control) × 100, A control is the absorbance of the control, which is composed of the DPPH radical solution + methanol (a mixture containing all reagents except the tincture) and A sample is the absorbance of DPPH radical + sample tincture. The percentage of DPPH decrease was expressed in Trolox equivalents (TE, R^2^ = 0.998). The DPPH radical scavenging activity of the tincture was expressed as IC50 (µg/mL). The assays were performed in triplicate [46,47,50].

#### 3.6.2. Ferric-Reducing Antioxidant Power Assay (FRAP)

The FRAP method is a spectrometric method that is based on the change of color of a complex of the 2,4,6-tri(2-pyridyl)-1,3,5-triazine (TPTZ) radical with the Fe^3+^ ion, which is assessed by the reduction of the ferric ion to the ferrous ion (Fe^2+^) in this complex [51]. The FRAP reagent is a mixture of 2.5 mL of a 10 mm TPTZ solution in 40 mM HCl, which are mixed with 2.5 mL 20 mm ferric chloride solution and 25 mL of acetate buffer at a pH of 3.6. Four mL of the tincture were diluted to 1.8 mL with water and mixed with 6 mL of the FRAP reagent. The blank solution was prepared similarly, using water instead of the tincture. The antioxidant capacity was assessed in correlation with the color change, by measuring absorbance at 450 nm. Trolox was used as a reference, using a calibration curve (R^2^ = 0.992). Results were expressed as µm Trolox equivalents/100 mL extract. The assays were performed in triplicate [52].

#### 3.6.3. Superoxide Radical (SO) Scavenging Activity Assay

The SO assay evaluated the ability of the tincture to inhibit the formation of the formazan by the reduction of the nitro blue tetrazolium (NBT) radical, by scavenging the superoxide radicals generated in the riboflavin-light-NBT system. The percentage of SO radical scavenging activity was calculated using the following formula: % Superoxide radical scavenging activity = (A_0_ − A_1_/A_0_) × 100, where A_0_ = absorbance of control (blank) and A_1_ = absorbance of tincture. The superoxide anion radicals (SO) were generated in a mixture of 2.0 mL of Tris-HCl buffer (16 mm, pH 8.0) with 2.0 mL of nitroblue tetrazolium (NBT, 0.3 mm) and 2.0 mL nicotinamide adenine dinucleotide solution (NADH, 0.936 mm). Then, 0.1 mL tincture was diluted to 100.0 mL with water and 1 mL of this solution was added to this mixture. Next, 2.0 mL phenazine methosulfate solution (PMS, 0.12 mm) were then added to all this to initiate the reaction and the mixture was incubated at 250 °C for 5 min. Absorbance was measured at 560 nm using a blank prepared from 2.0 mL Tris-HCl buffer, mixed with 2.0 mL NBT and 2.0 mL NADH solution, 4.0 mL water and 2.0 mL PMS solution. As a reference, 4.0 mL of 1.152 mg/mL Trolox solution was used and the results were expressed as µm Trolox equivalents/g. This solution was added to 2.0 mL Tris-HCl buffer, mixed with 2.0 mL NBT solution, 2.0 mL NADH solution and 2 mL PMS solution (0.12 mm). All tests were performed in triplicate [53].

#### 3.6.4. Animal Studies

The in vivo studies were conducted on outbreed Swiss mice, six months old, of 30.34 ± 2.87 g body weight. The animals originated from and were maintained throughout the study in the Establishment for Laboratory Animals of University of Agricultural Science and Veterinary Medicine Cluj Napoca. They were housed in conventional standard laboratory conditions (temperature 25 ± 1 °C, relative humidity 55 ± 5%, and 12 h light/dark cycle), five animals per cage. The animals benefited from environmental enrichment and had free access to granular standard food and water.

Housing conditions and the procedures complied with the Directive 2010/63/EU and national legislation, Law 43/2014. The project was approved by the Committee for Bioethics and Research Ethics of UASVM (accord No. 68/30.05.2017), and the Veterinary State Authorities (project authorization No. 73/14.06.2017).

Toxicity studies followed the Organization for Economic Cooperation and Development (OECD) Guidelines for the Testing of Chemicals, Test No. 425: Acute Oral Toxicity: Up-and-Down Procedure [54]. Five male and five female Swiss mice received a dose of 2000 mg/b.w. orally; all animals survived and showed no signs of toxicity. Fourteen days later, the animals were euthanized, then subjected to gross examination and displayed no alteration in the internal organs. Furthermore, liver and kidney showed a normal histological architecture.

The hepatoprotective effect was investigated using forty-five Swiss female mice. First, the animals were divided into five groups. Five animals were allocated to the control group, a group receiving placebo oral therapy (vegetable oil); that group was euthanized in the end of the study; they provided the reference values for plasma biochemistry and histopathology. The remaining forty animals were all subjected to liver insufficiency induction protocol; they received carbon tetrachloride (CCl_4_) in a dose of 1 mL/kg diluted in vegetable oil. CCl_4_ was administrated orally, three times a week, on days 1, 3 and 5, up to the end of the protocol, using a lubricated plastic probe. Those animals were divided into four equal groups; one group, the CCl_4_ group, received placebo therapy, and the remaining three groups were treated with *Rosmarinus officinalis* extract in doses of 5, 50 and 500 mg/kg b.w. The extract was administered on the same days as CCl_4_, four hours later. To prevent any influence of ethylic alcohol, before use, the alcoholic extract was maintained into a rotary evaporator until the entire amount of alcohol was removed, then it was reconstituted with distillate water and immediately administered to the animals. Each group of ten mice was further subdivided into two subgroups, five animals each, one was euthanized at four weeks, and the other was maintained for another two weeks.

In the end, the blood was collected from retroocular sinus, using deep isoflurane (3%) narcosis. The animals were considered dead when heart beats and respiratory movements stopped, the irreversibility of the phenomenon was assured by cervical dislocation, immediately followed by removal of internal organs. The blood was collected in clot activator vacutainers; after clotting, it was centrifuged (1465 g) for 15 min. The serum was immediately removed, stored at −20 °C and thawed just before use.

### 3.7. Oxidative Stress Markers and Plasma Biochemistry

In order to assess oxidative stress markers, extraction of total protein from liver tissue was done by homogenization with a phosphate buffer solution (at 10 mm, pH 7.4). The obtained protein extracts were analyzed for total protein content, catalase (CAT), superoxide dismutase (SOD) activity, glutathione peroxidase (GPx) activity, and lipid peroxidation. The activity of CAT, XOD and GPx was determined using the corresponding assay kits (BioVision, Milpitas, CA, USA), according to the manufacturer’s specifications. The concentration of malonyl dialdehyde (MDA) was determined using reaction with thiobarbituric acid (TBA). The results were measured by a METERTECH Spectrophotometer SP-830 Plus.

Serum chemistry was measured using screen point semi-automatic analyzer STAT-FAX 1904 Plus Global Medical Instrumentation Inc. (Ramsey, MN, USA) using special kits for plasma biochemistry (Diagnosticum Zrt. Hungary, Budapest) according to the producer specifications.

### 3.8. Histology

The tissue fragments were fixed in phosphate-buffered formalin 10%, pH 7, for 24 h, embedded in paraffin wax, cut to a thickness of 2–3 μm and stained with hematoxylin and eosin (H&E). The examination of the histopathological slides was performed using an Olympus image processing and retrieval system, the Olympus Cell B image acquisition and processing program. The histological examination was performed by an experienced pathologist RM, unaware of the therapy received by each group. Histopathological examination was focused on classic effects of CCl_4_ toxicity, such as circulatory, inflammatory, necrotic and fibrotic lesions.

### 3.9. Statistical Analysis

All data are reported as the mean ± SD (*n* = 5). To assume Gaussian distribution, normality distribution was checked by Shapiro–Wilk normality test two-way ANOVA, followed by Bonferroni post-test which was done for pairwise comparisons. Statistical significance was set at *p* < 0.05 (95% confidence interval). Statistical values and figures were obtained in GraphPad Prism version 5.0 for Windows, GraphPad Software, San Diego California USA.

## 4. Conclusions

The present study was conducted in order to offer a novel perspective on the species *R. officinalis*. The analyses that were performed on a fresh young shoots tincture highlighted the presence of polyphenols and terpenoids in *R. officinalis* fresh young shoots in significant amounts, showing, therefore, that the tested vegetal material may represent an important medicinal product. Moreover, this vegetal medicinal product was tested in order to evaluate its capacity to exhibit hepatoprotective activity by an antioxidant mechanism. Therefore, due to its chemical composition, the studied vegetal product belonging to the rosemary could be an important raw material for pharmaceutical formulations, contributing to the improvement of human health through its antioxidant and hepatoprotective properties, with significant effects against free radical damages, that may be useful for the protection against liver tissue damage.

## Figures and Tables

**Table 1:** Phenolic compounds identified in *R. officinalis* extract by HPLC–UV–MS.

Peak. No.	Compound	Structural Class	Retention Time R_t_ (min)	UV λ_max_ (nm)	[M + H]^+^ (m/z)	Concentration (μg/g d.w.)
1.	Syringic acid (3,5-Dimethoxy-4- hydroxybenzoic acid)	Hydroxybenzoic acid	3.30	265	198	355.84 ± 0.25
2.	Hesperidin (Hesperetin-rutinoside)	Flavanone	15.97	280	611	72.36 ± 0.15
3.	Nepetrin (Nepetin-glucoside)	Flavone	16.85	350, 265	479	259.65± 0.85
4.	Luteolin-glucuronide	Flavone	17.82	350, 260	463	698.70 ± 0.05
5.	Homoplantaginin (Hispidulin-glucoside)	Flavone	18.14	340, 260	463	364.31 ± 0.66
6.	Rosmarinic acid	Hydroxycinnamic acid	18.91	330	360	406.29 ± 0.95
7.	Luteolin-acetyl-glucuronide	Flavone	19.40	350, 260	505	146.82 ± 1.15
8.	Carnosol	Phenolic terpene	20.13	330	331	368.25 ± 0.33
9.	Luteolin	Flavone	21.74	350, 260	287	95.71 ± 1.02
10.	Nepetin	Flavone	21.89	350, 265	317	155.04 ± 0.98
11.	Rosmanol	Phenolic terpene	22.91	330	347	308.4 ± 1.02
12.	Rosmadial	Phenolic terpene	23.41	330	345	777.95 ± 0.85
13.	Cirsimaritin	Flavone	24.00	330, 260	315	713.7 ± 0. 96
14.	Carnosic acid	Phenolic terpene	25.39	270	332	804.27 ± 0.89

Note: Values represent the mean ± standard deviations of three independent measurements.

**Table 2:** GC–MS parameters for the main identified and quantified terpenoid compounds of *R. officinalis* tincture.

Terpenic Compound	Retention Time, min	Match Factor	Calibration Curve/R^2^	Detection (DL)/Quantification (QL) Limits, µg/mL	Content %(mg/g Tincture)
Camphene	6.73	832	A = 16.353·c + 193,775 R^2^ = 0.9971	DL = 23.7 QL = 47.4	0.34 ± 0.005
1,8-cineole	8.35	836	A = 13.712·c + 304.523 R^2^ = 0,9887	DL = 44.4 QL = 88.8	25.7 ± 0.308
Linalool	9.72	868	A = 13,796·c – 136,712 R^2^ = 0.9987	DL = 39.6 QL = 59.5	11.4 ± 0.148
Borneol	11.64	783	A = 14.380·c – 86.930 R^2^ = 0,98920	DL = 24.2 QL = 36.3	19.3 ± 0.212
Terpineol	12.19	865	A= 8324.8·c + 4,000,000 R^2^ = 0.9940	DL = 961 QL = 1922	1.9 ± 0.032

Values represent the mean ± standard deviations of three independent measurements.

**Table 3:** Polyphenols content and antioxidant activity of the fresh young shoots *R. officinalis* tincture.

Sample	TPC (mg GAE/g)	Flavonoid Total (mg RE/g)	Rosmarinic Acids (mg RAE/g)	DPPH (IC50, µg/mL)	FRAP (µm TE/g)	SO Scavenging (µm TE/g)
*R. officinalis* tincture	60.18 ± 0.42	33.01 ± 0. 24	25.40 ± 0. 84	31.85 ± 1.81	257.88 ± 1.74	99.70 ± 0. 65

Values represent the mean ± SD of three independent measurements. TPC = total polyphenols content; SO = superoxide anion radical; GAE = gallic acid equivalents; RE = rutin equivalents; RAE = rosmarinic acid equivalents; TE = Trolox equivalents.

**Table 4:** Composition of the GC-MS gradient of temperature.

Time	Temperature	Rate
0 min	80 °C	0 °C/min
7 min	220 °C	20 °C/min
11 min	240 °C	5 °C/min
24 min	240 °C	0 °C/min

## Data Availability

The data presented in this study are available on request from the corresponding author.

## Sample Availability

Samples of the compounds are not available from the authors.
